# Effect of RNA silencing suppression activity of chrysanthemum virus B p12 protein on small RNA species

**DOI:** 10.1007/s00705-020-04832-y

**Published:** 2020-10-10

**Authors:** Ramesh R. Vetukuri, Pruthvi B. Kalyandurg, Ganapathi Varma Saripella, Diya Sen, Jose Fernando Gil, Nina I. Lukhovitskaya, Laura J. Grenville-Briggs, Eugene I. Savenkov

**Affiliations:** 1 Department of Plant Breeding, Swedish University of Agricultural Sciences, Alnarp, Sweden; 2 Department of Plant Biology, Uppsala BioCenter, Swedish University of Agricultural Sciences, Linnean Center for Plant Biology, Uppsala, Sweden; 3 Department of Plant Protection Biology, Swedish University of Agricultural Sciences, Alnarp, Sweden; 4 Present Address: Division of Virology, Department of Pathology, University of Cambridge, Cambridge, UK

## Abstract

Chrysanthemum virus B encodes a multifunctional p12 protein that acts as a transcriptional activator in the nucleus and as a suppressor of RNA silencing in the cytoplasm. Here, we investigated the impact of p12 on accumulation of major classes of small RNAs (sRNAs). The results show dramatic changes in the sRNA profiles characterised by an overall reduction in sRNA accumulation, changes in the pattern of size distribution of canonical siRNAs and in the ratio between sense and antisense strands, lower abundance of siRNAs with a U residue at the 5′-terminus, and changes in the expression of certain miRNAs, most of which were downregulated.

**Electronic supplementary material:**

The online version of this article (10.1007/s00705-020-04832-y) contains supplementary material, which is available to authorized users.

RNA silencing has evolved as a widespread innate antiviral immunity mechanism in many eukaryotic organisms. Production of virus-derived small interfering RNAs (vsiRNAs) of 21, 22 and 24 nucleotides (nt) by host Dicer enzymes is an ubiquitous feature of any virus infection in plants [1]. vsiRNA are sorted into RNA-induced silencing complexes (RISCs), containing various Argonaute (AGO) proteins, based on the nucleotide residue at the 5′end to initiate the cleavage and destruction of cognate viral RNAs [2]. The major antiviral AGO proteins, namely AGO1 and AGO2, preferentially bind siRNAs with a U and A residue at the 5′ end, respectively [2–6]. Being the targets of RNA silencing machinery, plant viruses have evolved viral suppressors of RNA silencing (VSRs) that are able to dampen the host antiviral RNA silencing defence [7–9].

The cytoplasmically replicating chrysanthemum virus B – a member of the genus *Carlavirus* within the family *Betaflexiviridae* – encodes a multifunctional p12 protein. Previously, we showed that the nuclear-localised fraction of the protein is involved in activation of transcription of certain genes, developmentally reprogramming the host for the benefit of the virus [10]. P12 also acts as a VSR in the cytoplasm [11], yet much work remains to be done to better understand the mechanisms of RNA silencing suppression by p12. The first step towards this goal would be the characterisation of small RNA (sRNA) profiles during p12 expression, and comparison of those to appropriate negative controls as well as to sRNA profiles obtained upon expression of a well-characterised VSR, *e*.*g*., HcPro of potyviruses.

For the past decade, high-throughput sequencing (HTS) has become a widespread method to characterise sRNAs associated with viral infections or to compare small-RNA profiles in the presence and absence of VSR expression [12–14]. However, most of these studies have focused on rather late stages of virus infection, when the virus is readily detectable in the plant (1-2 weeks post-inoculation). Here, we sought to characterise sRNAs 96 h after expression of the VSR, thus, mimicking the early stages of the infection process.

P12 is able to suppress RNA silencing by complementing a turnip crinkle virus lacking a VSR gene (TCV-sGFP) [11]. However, until now, there has been no analysis of sRNAs in the presence of p12. Thus, there is a need to determine whether and which sRNAs are targeted by p12. Furthermore, comparison of sRNA profiles between a weak (p12) and strong (HcPro) VSR could provide information as to the possible mechanisms of RNA silencing suppression. The effect of p12-mediated silencing suppression on small RNA accumulation was investigated by performing HTS of small RNAs. To this end, *Nicotiana benthamiana* plants were grown under long day conditions (16h light/8h dark) in growth chambers with a minimum daytime temperature of 20 °C and a night-time temperature of 18 °C. Fully-expanded leaves of 1-month-old *N. benthamiana* plants were agro-infiltrated for co-expression of *GFP* plus empty plasmid control (GFP/EP), or *GFP* plus potato-virus-A-encoded HcPro (GFP/HcPro), or *GFP* plus p12 (GFP/p12) (Supplementary Fig. 1). For each treatment, we used three plants (three biological replicates) with two leaves being infiltrated on each plant. Tissues from these leaves were collected prior RNA extraction.

Total RNA was isolated using a mirVana miRNA Isolation Kit (Ambion) according to the manufacturer’s instructions, and the RNA quality was assessed with the aid of Agilent Bioanalyzer chips. Nine sRNA libraries were constructed using total RNA isolated from infiltrated patches of *N. benthamiana* leaves 4 days post-infiltration (dpi) and a TruSeq RNA Library Preparation Kit (Illumina, Inc.). Sequencing was performed on an Illumina HiSeq2500 instrument in high-output mode, with single reads of 1 × 50 bp at SciLifeLab, Stockholm. Quality assessment of raw reads was performed using FastQC v 0.11.3. Adapter trimming was carried out using cutadapt v 1.2.1 with a minimal sequence length of 18 after adaptor removal [15]. Cleaned reads from each library were aligned to *N. benthamiana* reference sequences or *sGFP* using Bowtie v1.2.2, allowing two mismatches per seed (-n 2), a seed length of 18 (-l 18), and no mismatch in the read alignment (-v 0) and with the rest of the alignment parameters set to their default values. SAM alignment files produced by Bowtie were converted to BAM files, sorted, and indexed using SAMtools v 0.1.19 [16]. Counts of reads per sRNA length class were obtained from each library. Additionally, sense/antisense location, position along the reference sequence, and nucleotide preference for each length class were determined with SAMtools and custom scripts written in bash and perl. Read counts for each library were normalized into reads per million (RPM) and averaged across replicates. Differential expression analysis of annotated *N. benthamiana* miRNAs [17] was carried out with Salmon and DESeq2. Briefly, miRNA sequences were indexed and quantified using Salmon v 0.9.1, and differential expression analysis was carried out using DESeq2 (Supplementary Table 1). Graphs showing counts of sRNA along reference genes were plotted in R v 3.5.1. All other graphs were plotted in Excel v 16.16.15.

Sequencing of nine sRNA libraries resulted in 12,370,110 to 35,127,860 cleaned high-quality reads for each sample (Table 1). As shown in Table 1, the abundance of *GFP*-derived sRNAs was as high as 16.4% in the GFP/EP library, whereas it was below 9.6% in the GFP/HcPro libraries, or even below 3.7% in the GFP/p12 libraries (Table 1; Fig 1a).

**Table 1:** Summary of the results of NGS of small RNAs from wt *Nicotiana benthamiana* leaves co-expressing GFP with either EP or HcPro or p12

Library	Replicate	Total reads^a^	Reads after QC	HQ reads	Reads mapped to *N. benthamiana* genome	Percentage of reads mapped to *N. benthamiana* genome	Reads mapped to GFP	Percentage of reads mapped to GFP
GFP/EP	Replicate 1	35,207,536	22,128,179	62.85%	12,204,784	55.15%	3,627,840	16.39%
	Replicate 2	31,377,982	17,199,648	54.81%	9,578,609	55.69%	2,818,900	16.39%
	Replicate 3	29,536,107	16,121,007	54.58%	9,010,874	55.90%	2,644,477	16.40%
GFP/HcPro	Replicate 1	16,832,919	12,370,110	73.49%	6,100,105	49.31%	1,046,859	8.46%
	Replicate 2	48,556,722	35,127,860	72.34%	17,050,815	48.54%	3,385,821	9.64%
	Replicate 3	26,259,510	17,532,394	66.77%	8,534,076	48.68%	1,651,377	9.42%
GFP/p12	Replicate 1	22,489,465	14,900,009	66.25%	7,779,266	52.21%	555,643	3.72%
	Replicate 2	24,050,417	14,699,018	61.12%	8,042,616	54.72%	505,232	3.44%
	Replicate 3	26,191,281	16,539,121	63.15%	9,092,689	54.98%	526,344	3.18%

**Fig. 1:**
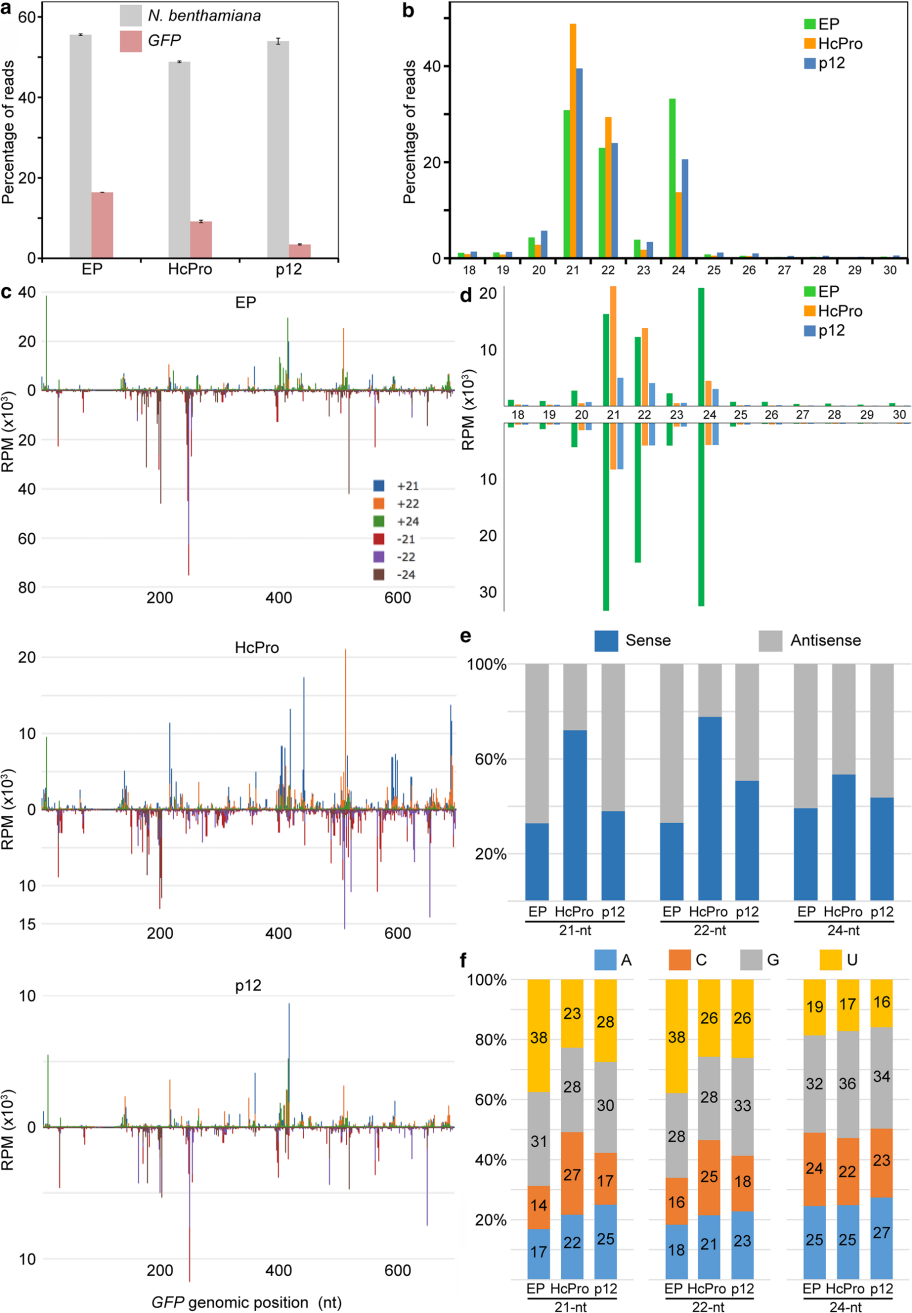
Profiles of *GFP*-derived small interfering RNAs (siRNAs) captured by deep sequencing from wt *Nicotiana benthamiana* leaves co-expressing *GFP* with either EP or *HcPro* or *p12*. (**a**) Percentage of total small RNAs (sRNAs) reads (18-30 nt) derived from the nine libraries. **(**b) Size distribution of 18- to 30-nt sRNAs mapped to the *GFP* gene. (**c**) Representative images of single-nucleotide resolution maps of siRNAs aligned to the *GFP* gene. Peaks of different colours indicate three classes (21-, 22- and 24-nt) of GFP-specific siRNAs derived from sense and antisense strands. (**d**) Size distribution and strand polarity of 18- to 30-nt GFP-derived siRNAs. The graphs show read count (abundance) per million mapped reads. (**e**) Percentage of sense and antisense *GFP*-derived siRNAs. (**f**) Relative frequency of the 5′-terminal nucleotide of *GFP*-derived siRNAs. The data represent averages of three libraries for each treatment. RPM, reads per million

The sRNA read length analysis demonstrated that the sizes of siRNAs from all three treatments centred at 21, 22 and 24 nt (Fig. 1b; Supplementary Fig. 2). The general size distribution of the sRNA reads was in line with previous reports [14, 18]. However, the normalized size distribution of total sRNAs differed between the treatments, with 24-nt species being predominant (33.2%), followed by 21-nt and 22-nt sRNA populations in the GFP/EP library (Fig. 1b). On the other hand, the pattern of size distribution was different in the presence of HcPro and p12, with 21-nt species being more prevalent (48.8% and 39.5%, respectively) than 22-nt and 24-nt sRNA populations (Fig. 1b).

Further analyses were performed by aligning canonical sRNAs of the 21-nt, 22-nt and 24-nt size classes to the sGFP sequence (Fig. 1c). The single-nucleotide resolution maps generated for each treatment showed that the polarity of siRNA reads from different treatments displayed huge variations (Fig. 1c). The prevalence of antisense (60.8-67.1%) over sense-strand siRNAs (32.9-39.2%) was observed in the GFP/EP libraries (Fig. 1d and e), suggesting efficient incorporation of the antisense strand of siRNAs into RISC. Here, they mediate cleavage of GFP mRNA by means of complementarity and, at the same time, get stabilised and protected from rapid degradation, whereas the corresponding ‘passenger’ sense strand gets cleaved/degraded. In contrast, in the GFP/HcPro libraries, sense strands of siRNAs were more abundant (sense, 53.5-77.8% *versus* antisense, 22.2-46.5%) for all canonical sRNAs of the 21-nt, 22-nt and 24-nt size classes (Fig. 1d and e). Thus, expression of HcPro markedly enhances the GFP siRNA bias toward the sense strand (Fig. 1e). This can be explained by the previously reported ability of HcPro to sequester siRNA through size-specific binding and to interfere with the methylation of siRNA. Thus, the stability of siRNAs is affected [19–21]. Similar amounts of sense and antisense 22-nt and 24-nt siRNAs were found in the GFP/p12 libraries (Fig. 1e), whereas the ratio of sense to antisense of 21-nt siRNAs was similar to the EP control samples (37.9% to 62.1%; Fig. 1e), suggesting little (or no) effect on 21-nt species on p12 expression. Several siRNAs hot spots were detected on the GFP transcript (Fig. 1c). Most of these hot spots clustered toward the 3′-proximal region, suggesting involvement of host RNA-dependent RNA polymerase 6 (RDR6) in converting the GFP transcript into dsRNA that is then cleaved into siRNAs. In the presence of VSRs, 21-nt and 22-nt siRNAs were predominant within these hot spots, whereas 24-nt siRNA, followed by 21-nt and 22-nt siRNAs, predominated within hot spots in the GFP/EP libraries (Fig. 1c).

Selective loading of sRNAs into specific AGOs is preferentially directed by the 5′-terminal nucleotide [2]. In light of this information, we chose to more closely examine the relative abundance of sRNAs according to the nucleotide residue at their 5′-terminus. To assess the base preference at the 5′ end, we calculated the relative frequency of each of four bases, which was then normalised by the average percentage of each base along the selected positions on the reads. We found that 21- and 22-nt siRNAs size classes with U residue at the 5′ end were less abundant (22.7-27.5%) in the GFP/HcPro and GFP/p12 libraries than in the GFP/EP control (37.5-37.9%), whereas there was no effect on the 24-nt siRNA size class (Fig. 1f). The base C was the least favoured at the 5′ end of 21- and 22-nt siRNAs size classes for the EP control (16.8 and 18.3%, respectively) and p12 (17.3 and 18.5%, respectively), whereas this bias shifted to an A residue for HcPro (21.6 and 21.5%, respectively; Fig. 1f).

Differentially expressed miRNAs (DEMs) were obtained using the approach described above. In total, the expression of 53 and 55 *N. benthamiana* miRNAs could be measured in GFP/p12- and GFP/HcPro-infiltrated leaves, respectively (Supplementary Fig. 3, Supplementary Table 1). After filtering (*p* < 0.05), nine miRNAs were differentially expressed in the GFP/p12 dataset, and five miRNAs in GFP/HcPro dataset (Fig. 2a, bars with asterisks; Supplementary Fig. 3, Supplementary Table 1). Only one miRNA (miR479) was differentially expressed in both datasets (Fig. 2a). Thus, a total of 13 DEMs were obtained for the VSR groups (Fig. 2a). To confirm the sRNA-seq results, the abundance levels of these 13 miRNAs were tested using a quantitative stem-loop RT-qPCR [22] (Fig. 2b). A regression analysis showed a very weak positive correlation (Pearson’s correlation coefficient, r = 0.20) between the log_2_ fold change in the HTS data and quantification of the miRNAs by RT-qPCR (Fig 2a and b; data not shown). Interestingly, according to the stem-loop RT-qPCR, most of the miRNAs analysed (9 out of 13) were downregulated in the presence of p12, whereas six miRNAs were upregulated and seven were downregulated upon HcPro expression (Fig. 2b).

**Fig. 2:**
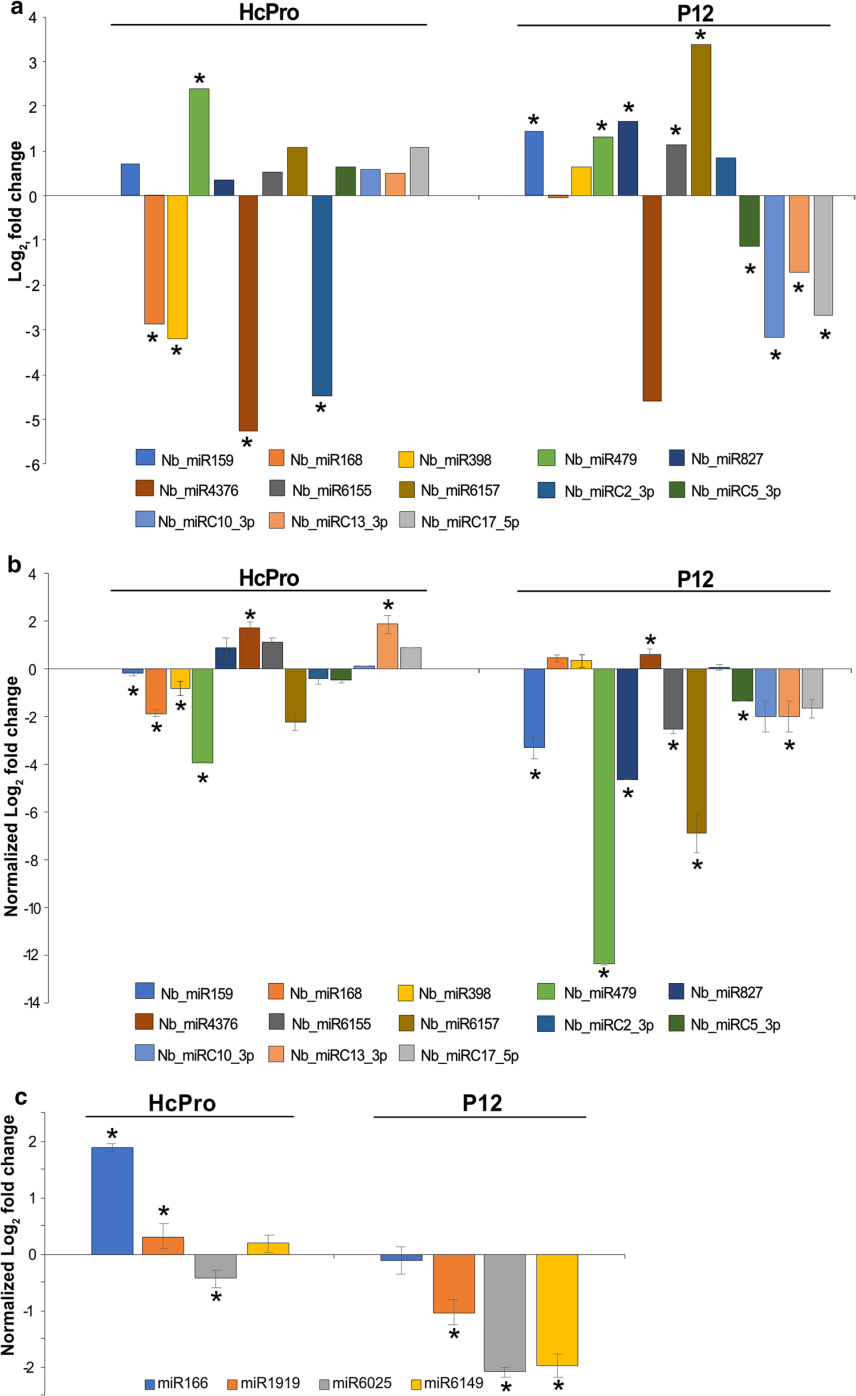
Quantification of miRNAs. (**a**) Log_2_ fold change of deferentially expressed miRNAs (DEMs) identified in the HTS data after filtering (*p* < 0.05). (**b**) Validation of HTS data by stem-loop RT-qPCR. Log_2_ relative fold expression miRNAs identified as DEMs in the HTS data. (**c**) Log_2_ relative fold expression of three miRNAs predominant in *Solanaceae* species and a highly conserved miR166. The expression levels were normalised to the expression of *NbPP2A*, and EP control set to value of 1. Asterisks indicate significant statistical differences relative to EP control, *p < 0.05; Student’s two-tailed *t*-test

Additionally, we chose to verify expression levels of four miRNA using a quantitative stem-loop RT-qPCR. These miRNAs included miR166 belonging to a highly conserved miRNA family and three miRNAs predominant in Solanaceae species (miR1919, miR6025 and miR6149) [17]. The analysis showed upregulation of expression of miR166 in the presence of HcPro, but not p12 (Fig. 2c). On the other hand, miR1919 and miR6149 were strongly downregulated upon expression of p12, but slightly upregulated by HcPro. MiR6025 was downregulated in the presence of both HcPro and p12 (Fig. 2c), although the relative level of reduction of miR6025 was greater with p12 expression (4.35-fold on average) than with HcPro expression (1.35-fold on average).

Using global sRNA-seq and real-time PCR analysis, we identified some differences in the accumulation of certain classes of siRNAs and miRNAs in the presence of VSRs analysed in this study. These differences included (i) an overall reduction in the number of GFP sRNA reads in the presence of VSRs relative to the EP control (~3.2-9.6% versus ~16.4%), (ii) a shift in the prevalence of canonical sRNA, with 24-nt siRNA being most abundant in the absence of VSR expression and 21-nt species being more abundant when VSRs were expressed, (iii) changes in the ratio between sense and antisense strands of siRNA with the prevalence of antisense over sense strand in the absence of VSR expression, whereas sense-strand siRNA were more abundant in the presence of HcPro and almost equal amounts of sense and antisense strands of the 22-nt and 24-nt size classes, but not 21-nt siRNAs, were present upon p12 expression, and (iv) lower abundance of siRNAs with a U residue at the 5′ terminus upon expression of both HcPro and p12 as compared to EP control. Supporting these observations, we recently showed that a weak VSR, the 8K protein, encoded by a P1 isolate of a soil-borne potato mop-top virus (PMTV) interferes with accumulation of certain classes of sRNAs [14]. Notably, the accumulation of anti-sense strands of 22-nt siRNAs was reduced, and the proportion of siRNAs with a 5′-terminal nucleotide residue U was lower in the GFP/8K libraries than in the GFP/EP libraries [14]. The higher reduction in antisense than sense virus-specific siRNA was also observed in *NbRDR6-*silenced plants infected with a soil-borne Chinese wheat mosaic virus (CWMV) [12]. Interestingly, it has also been reported that the proportion of siRNAs with a U residue at the 5′ terminus in plants infected with CWMV was lower at low (16 °C) temperatures than in those infected at high (24 °C) temperatures [12]. Considering that siRNAs with a 5′-terminal U are mostly loaded into AGO1, an AGO with a major antiviral function [3–6], it is possible that a smaller proportion of siRNA are incorporated into the RISC at low temperatures or upon expression of HcPro, 8K, or p12 VSRs, thus dampening the antiviral response under these conditions.

Overall, our data show dramatic changes in sRNA profiles already after 96 hours of VSR expression with a clear difference between profiles generated for HcPro (a strong VSR) and p12 (a weak VSR). Further experimentation on the impact of p12 on cellular RNA silencing pathways may be a fruitful line of inquiry in the future.

## Electronic supplementary material

Below is the link to the electronic supplementary material.

Supplementary material 1 (PDF 935 kb)

Supplementary material 2 (XLSX 16 kb)

## Abbreviations

AGO: Argonaute
CVB: Chrysanthemum virus B
CWMV: Chinese wheat mosaic virus
DEM: Differentially expressed miRNA
GFP: Green fluorescent protein
PMTV: Potato mop-top virus
miRNA: Micro RNA
NGS: Next-generation sequencing
RDR6: RNA-directed RNA polymerase 6
RISC: RNA-induced silencing complex
RPM: Reads per million
siRNA: Small interfering RNA
sRNAs: Small RNAs
vsiRNAs: Virus-derived small interfering RNAs
VSR: Viral suppressor of RNA silencing
