# Supplementary material for: Effect of RNA silencing suppression activity of chrysanthemum virus B p12 protein on small RNA species
Source: Arch Virol. 2020 Oct 10;165(12):2953–9. doi: 10.1007/s00705-020-04832-y (PMC7588395; doi:10.1007/s00705-020-04832-y)
Supplement: Supplementary file 1 — Supplementary material 1 (PDF 935 kb) [file 705_2020_4832_MOESM1_ESM.pdf]

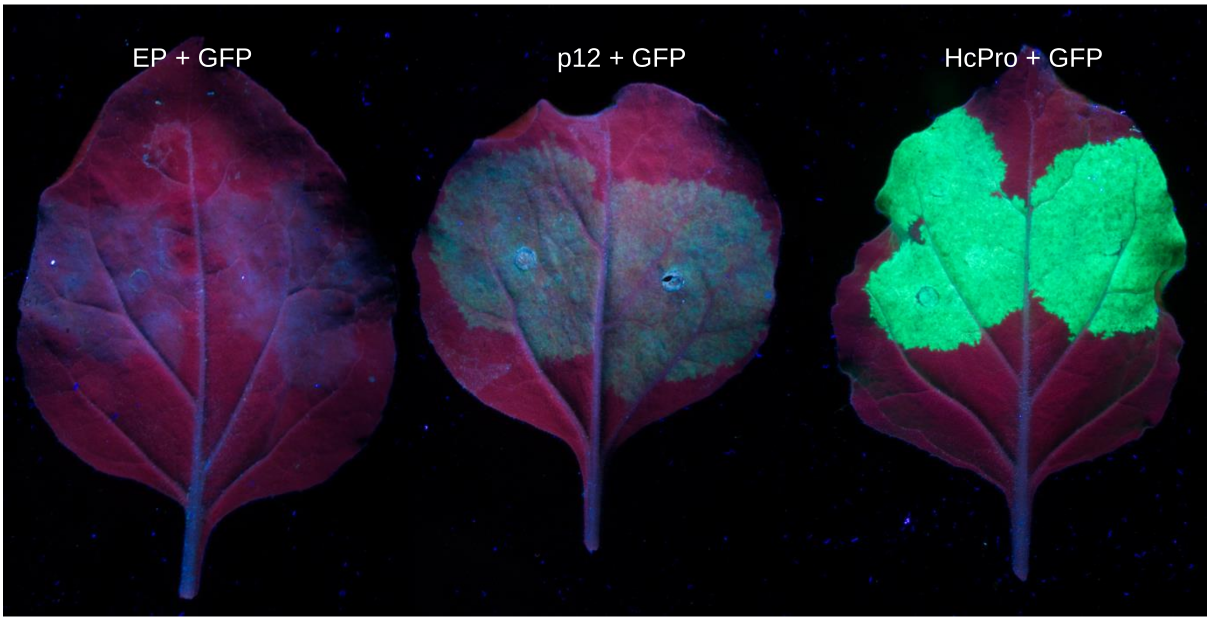

Supplementary Figure 1. P12 suppresses RNA silencing triggered by GFP in an agroinfiltration assay in *Nicotiana benthamiana* leaves. Infiltration of a leaf was carried out at two spots for expression of empty plasmid control (EP; left), p22 (middle), or HcPro (right) together with GFP. The picture was taken 4 days post-infiltration with Nikon D90, with a constant exposure time of 8s at f/20.

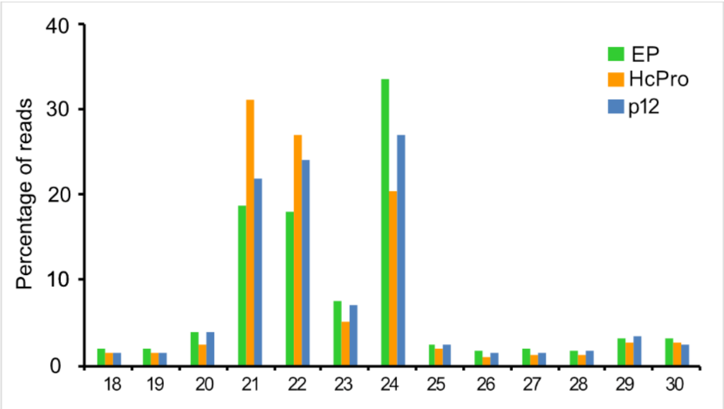

Supplementary Figure 2. Size distribution of 18-30 nt small RNAs mapped to *Nicotiana benthamiana* genome.

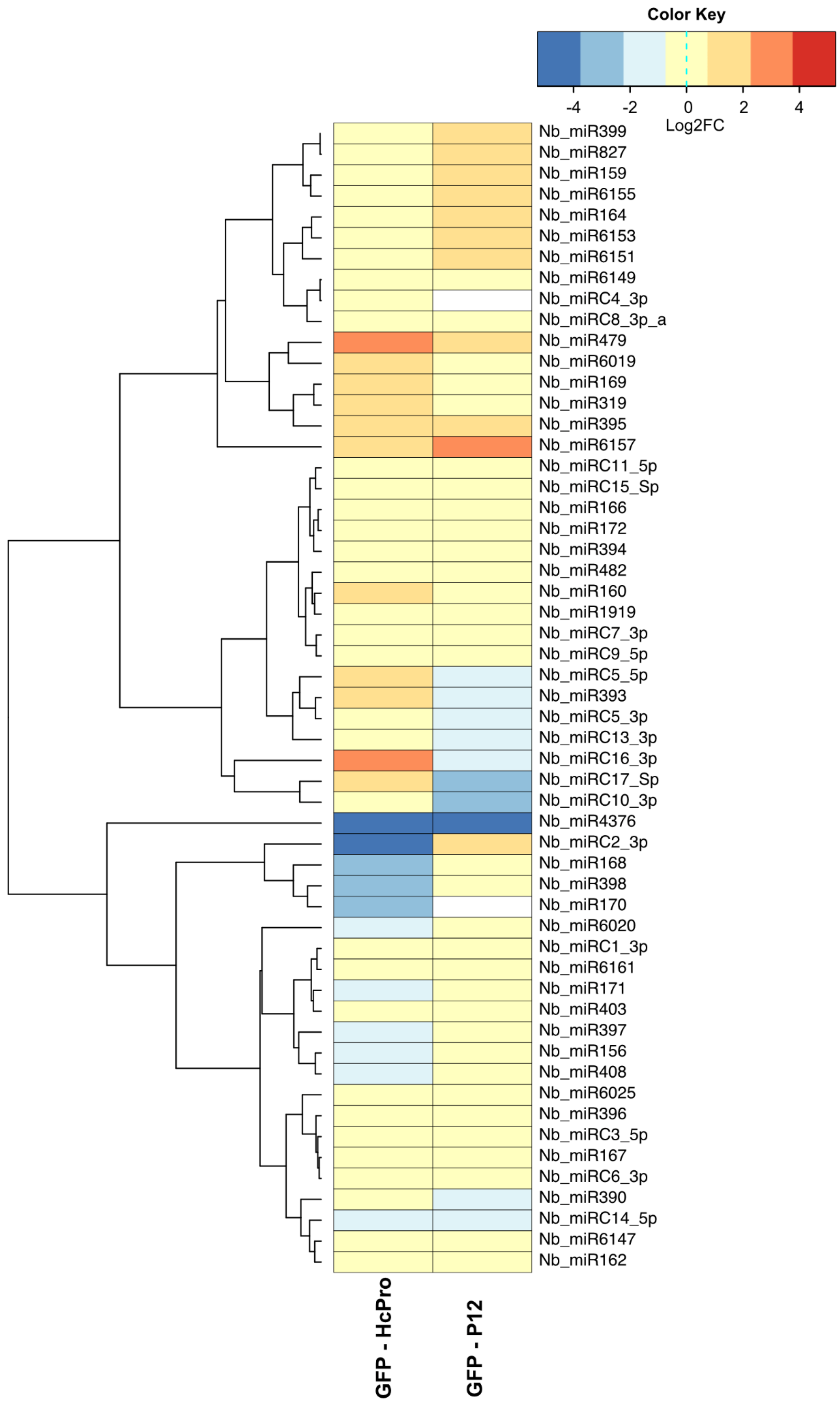
Color Key
-4
-2
0
2
4
Log2FC
Nb_miR399
Nb_miR827
Nb_miR159
Nb_miR6155
Nb_miR164
Nb_miR6153
Nb_miR6151
Nb_miR6149
Nb_miRC4_3p
Nb_miRC8_3p_a
Nb_miR479
Nb_miR6019
Nb_miR169
Nb_miR319
Nb_miR395
Nb_miR6157
Nb_miRC11_5p
Nb_miRC15_Sp
Nb_miR166
Nb_miR172
Nb_miR394
Nb_miR482
Nb_miR160
Nb_miR1919
Nb_miRC7_3p
Nb_miRC9_5p
Nb_miRC5_5p
Nb_miR393
Nb_miRC5_3p
Nb_miRC13_3p
Nb_miRC16_3p
Nb_miRC17_Sp
Nb_miRC10_3p
Nb_miR4376
Nb_miRC2_3p
Nb_miR168
Nb_miR398
Nb_miR170
Nb_miR6020
Nb_miRC1_3p
Nb_miR6161
Nb_miR171
Nb_miR403
Nb_miR397
Nb_miR156
Nb_miR408
Nb_miR6025
Nb_miR396
Nb_miRC3_5p
Nb_miR167
Nb_miRC6_3p
Nb_miR390
Nb_miRC14_5p
Nb_miR6147
Nb_miR162
GFP - HcPro
GFP - P12

Supplementary Figure 3. Heat map of the expression of 55 *Nicotiana benthamiana* miRNAs mesured by high-throughput sequencing of GFP/HcPro and GFP/p12 libraries as comapred to GFP/EP control.
